# Mining Differentially Expressed Genes in the Marine Free-Living Flatworm *Macrostomum lignano* Under Aneuploidy-Driven Ploidy Changes

**DOI:** 10.3390/cells15030245

**Published:** 2026-01-27

**Authors:** Kira S. Zadesenets, Nikita I. Ershov, Natalya P. Bondar, Konstantin E. Orishchenko, Nikolay B. Rubtsov

**Affiliations:** 1 Institute of Cytology and Genetics, Russian Academy of Sciences, 630090 Novosibirsk, Russia; nikotinmail@mail.ru (N.I.E.); nbondar@bionet.nsc.ru (N.P.B.); orishchenkoke@icg.sbras.ru (K.E.O.); rubt@bionet.nsc.ru (N.B.R.); 2 Laboratory of Structural and Functional Organization of the Genome, Novosibirsk State University, 630090 Novosibirsk, Russia

**Keywords:** flatworms, aneuploidy, polyploidy, whole-genome duplication, differentially expressed gene, transcriptome, gene expression, subgenomic organization, trans-effects

## Abstract

Whole-genome duplication (WGD) is a powerful evolutionary force, yet the mechanisms by which neopolyploids achieve transcriptomic stability and phenotypic success remain poorly understood. This study investigated the phenotypic and transcriptomic consequences of ploidy changes in the flatworm *Macrostomum lignano*, a “successful” neopolyploid model. We exploited two established sublines derived from the inbred DV1 line: the euploid DV1_8 (hidden tetraploid, SSL_1_L_2_) and the aneuploid DV1_10 (hidden hexaploid, SSL_1_L_1_L_2_L_2_). By integrating whole-genome sequencing (WGS)-informed normalization with RNA-seq analysis, we differentiated true regulatory shifts from gene-dosage effects. We revealed that while most genes scale linearly with ploidy, 1308 genes exhibited a nonlinear aneuploidy-induced transcriptional response. The remarkable trans-acting effects were observed across subgenome S encoded by disomic small chromosomes. Differentially expressed genes (DEGs) were enriched in pathways essential for homeostasis and growth: mTOR signaling, ubiquitin-mediated proteolysis, and the Hippo/Wnt pathways. Phenotypes of the DV1_10 worms exhibited increased body size, enhanced cell proliferation, and higher viability in comparison to the DV1_8 worms (60.25% vs. 21.5%). These findings suggest that *M. lignano* possesses mechanisms for dosage compensation to mitigate the deleterious effects of aneuploidy. Ultimately, this study demonstrates how genomic plasticity and rewiring of the transcriptome may facilitate the evolutionary success of animal neopolyploids.

## 1. Introduction

Gene duplication is one of the major evolutionary forces, providing the acquiring of new genetic material that potentially can also serve as a new platform for the emergence of genetic novelty in evolution [1]. It can result from small-scale events like unequal crossing over [2], retroposition [3], or whole-genome duplication (WGD) [4]. Considering the evolutionary fates of the duplicated gene copies, they undergo pseudogenization (or loss) and sub- and neofunctionalization [5]. Paralogs characterized by only small differences are similar to gene alleles, but they cannot be lost as a result of inbreeding. The WGD is more dramatic in comparison with gene duplication: a polyploid genome is often highly dynamic and is subject to numerous changes [6]. Furthermore, polyploids can exhibit wide variation in phenotypes that are not found in their diploid ancestors [7]. Moreover, new transcriptomic patterns in polyploid species can differ from the ones of their diploid ancestor(s), leading to transcriptomic and phenotypic novelty [8]. In plants, comparison of gene expression in di- and polyploid species revealed considerable differences, resulting in overall new phenotypic traits in polyploids. For instance, plants have achieved enhanced tolerance to stress conditions such as drought, salinity, and extreme temperatures through genome buffering, increased flexibility of gene expression, and epigenetic modifications [9]. The increased adaptability is considered an evolutionary advantage under environmental changes.

The consequences of the WGD are complex and can differ among different species. Despite the large genetic diversity, which can be generated by the WGD and further genome reorganization, only a few WGD events are evolutionarily ‘successful.’ The contemporary successful polyploids are only a small part of the arisen polyploid forms, and their phenotypic variation had the advantageous traits to be favored for selection of the existing polyploid form among many other unobserved or extinct variants [7]. The studies of modern neopolyploids are essential for understanding the underpinning reasons for their evolutionary success.

*Macrostomum lignano*, a free-living marine flatworm, is one of the astonishing neopolyploid ‘successful’ species. It has served as an exciting model organism for studies on numerous biological processes and mechanisms of development, regeneration, aging, sex allocation, bio-adhesion, karyotype evolution, etc. [10,11,12,13,14,15,16,17,18,19,20,21,22].

We uncovered the hidden polyploidy of the *M. lignano* genome masked with intensive karyotype reorganization after the recent WGD [16,23,24,25,26]. The karyotype of *M. lignano* consists of four chromosome pairs [27]; one pair consists of the large chromosomes (chromosome L), while the others are three pairs of small metacentrics (chromosomes S). The chromosome L contains highly homologous paralogous regions to nearly all regions of the S chromosomes [21,25,26], indicating that cytogenetic rediploidization after WGD included the fusion of small ancestral chromosomes into the large one followed by its intensive rearrangement. Furthermore, the chromosome rearrangements led to the differentiation and genetic isolation of the L chromosome copies (L_1_ and L_2_). As a result, nearly all the *M. lignano* genes from the specimens of the inbred DV1 line have three paralogs; two of them were encoded with genes located in the different two copies of the L chromosome, while one was encoded with a gene located in the S chromosome. It allowed isolating three subgenomes in the *M. lignano* genome. Two of these are associated with copies of the L chromosome (L_1_ and L_2_) as haploid ones, while the highly homozygous subgenome associated with the S chromosome is the diploid one. Such unusual genome organization (SSL_1_L_2_) facilitated preservation of the high genetic diversity in *M. lignano*, even in the highly inbred laboratory line DV1 [21].

The previously established two laboratory sublines of the worms originated from the DV1 line: one subline, DV1_10, with two additional copies of the large chromosome (2n = 10; SSL_1_L_1_L_2_L_2_), and another subline, DV1_8, consisted of the euploid worms (2n = 8; SSL_1_L_2_) [21]. Formally, worms of the DV1_10 subline are aneuploids, but it would more correct to consider them as hidden hexaploids, while euploid worms of the DV1_8 are hidden tetraploids. To maintain the balance of gene products, the expression level of most genes scales linearly with an increase in the genome ploidy; however, specific dosage-sensitive genes may respond disproportionally [28].

The present study extends our earlier work on these sublines [21] by transitioning from genomic description to a detailed transcriptomic analysis of the DV1_8 and DV1_10 sublines. In this study, we sought to identify these nonlinear transcriptomic changes and evaluate the resulting phenotypic variation between the DV1_8 and DV1_10 worms. By integrating WGS-informed normalization with RNA-seq analysis, we characterized the aneuploidy-induced transcriptional response, specifically investigating how *M. lignano* manages the transition between tetraploidy and hexaploidy at the transcriptional level.

## 2. Materials and Methods

### 2.1. Study Organism

**Figure 1:**
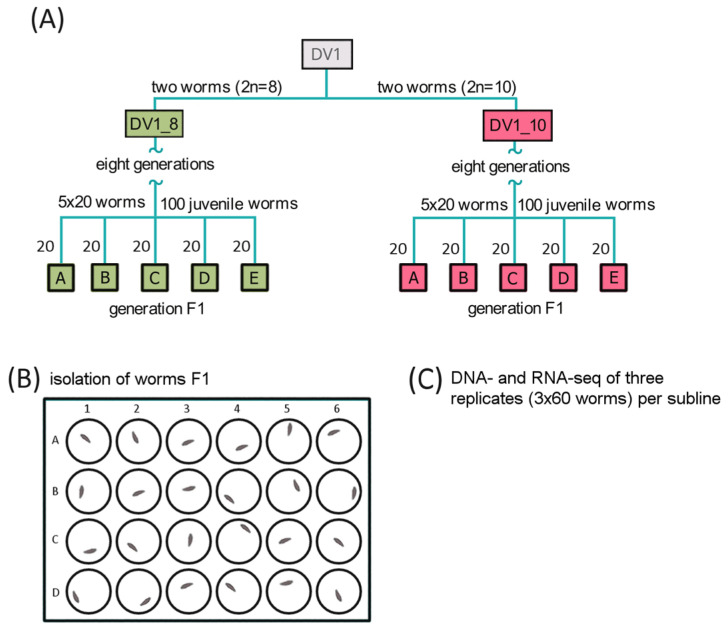
Experimental design for the DNA- and RNA-seq analyses of the DV1_8 and DV1_10 sublines [21]. (**A**) Generation of the sublines DV1_8 and DV1_10 and following generation of worm pools DV1_8A-E and DV1_10A-E. (**B**) Isolation of the hatchlings from F1 in pools in 24-well plates. (**C**) Obtaining replicates of 60 adult F1 worms of the same age from pools A–E. Selection of the worm pools (in total three replicates per each subline) for following whole-genome and transcriptome sequencing.

The commonly used inbred line of *M. lignano*, DV1 has been established via full-sib and half-sib inbreeding for 24 generations [29] and has been maintained under standard laboratory conditions [13] at small population sizes for a high level of genetic homozygosity. For a few years, the inbred DV1 line was regularly karyotyped, and a high karyotype instability associated with the variable copy number of the largest chromosomes was revealed [16,24,25]. Previously, we established purebred sublines with two different karyotypes, namely (i) the species-specific 2n = 8 karyotype of *M. lignano* and (ii) the 2n = 10 karyotype with four copies of the largest chromosome. The obtained sublines were characterized with the stable karyotype. The experimental design was described earlier [21]. Briefly, we chose three out of five worm pools for each subline, DV1_8 and DV1_10 (DV1_8A, DV1_8C, and DV1_8E and DV1_10A, DV1_10C, and DV1_10E, respectively) [21] according to the data on reproduction, mortality, morphology, and/or behavior abnormality (Figure 1). We generated two replicate libraries for whole-genome sequencing from each of the selected pool; each library was generated from 60 worms. For transcriptome analysis of intact adult worms, we established three biological replicates generated from the worm pools DV1_8A, DV1_8C, and DV1_8E for the DV1_8 subline and three biological replicates generated from the DV1_10A, DV1_10C, and DV1_10E worm pools, respectively, for the DV1_10 subline.

### 2.2. RNA Extraction, Library Preparation, and RNA Sequencing

The total RNA was extracted from pooled worms using PureZOL (Bio-Rad, Hercules, CA, USA), according to the manufacturer’s instructions. The samples were treated with DNase I (New England Biolabs, Ipswich, MA, USA) to eliminate DNA contamination. The total RNA was purified on Agencourt RNAClean XP beads (Beckman Coulter, Krefeld, Germany). The quantity of each sample was assessed using Qubit 2.0 (Life Technologies, Carlsbad, CA, USA).

RNA-seq libraries were prepared in accordance with New England Biolab protocols. Briefly, polyA-tailed mRNA was purified from 300 ng of total RNA using the NEBNext Poly(A) mRNA Magnetic Isolation Module (New England Biolabs, E7490S). Then, directional cDNA libraries were generated using the NEBNext Ultra II Directional RNA Library Prep Kit for Illumina (New England Biolabs), following the manufacturer’s recommendations, and index codes were added to attribute sequences to each sample. The clustering of the index-coded samples was performed on a cBot Cluster Generation System using TruSeq PE Cluster Kit v4-cBot-HS (Illumina, San Diego, CA, USA), according to the manufacturer’s instructions. After cluster generation, the library preparations were sequenced on an Illumina HiSeq 2500 platform, and paired-end reads were generated. The libraries’ quality was assessed on the Agilent Bioanalyzer 2100 system (Agilent Technologies, Inc., Santa Clara, CA, USA) (Figures S1 and S2).

### 2.3. Alignment and Assembly of RNA-Seq Data and Update of the Reference Gene Annotation

The bulk RNA-seq data obtained during the project (the entire dataset, approximately 240 million paired reads) were used to create an expanded gene annotation of the *M. lignano* reference genome assembly. The main goals to be achieved by creating an updated *M. lignano* transcriptome annotation were that all obtained transcripts should be present in the assembly and all gene paralogs should be annotated. RNA-seq data were preprocessed by cutadapt to remove adapters and SL exons and aligned to the existing *M. lignano* reference genome assembly version Mlig_3_7 (GenBank accession: GCA_002269645.1) [30], using the STAR mapping v.2.7.9a two-mapping strategy [31]. To generate the annotation, genome-guided transcriptome assemblies were obtained from mapped RNA-seq data (StringTie v2.1.7b, PsiCLASS v1.0.3, and TransMeta v.1.0), and reliable splice sites were annotated using Portcullis v1.2.4 [32] on the reference genome. Furthermore, using minimap v.2.24-r1122, mRNAs and CDSs from the reference annotation Mlig_3_7 (macrostomum_lignano.PRJNA371498.WBPS14.annotations.gff3) were remapped with multiple matches allowed. The obtained data were integrated into a consensus gene annotation using Mikado v2.3.4 [33] with the original Mlig_3_7 annotation set as reference and filtering parameters optimized for flatworms.

The resulting and original reference annotations were compared using gffcompare v0.10.5 [34]. As a result, 48,604 of the 63,161 genes in the new annotation shared a common location with the Mlig_3_7 reference genes. Thus, the update to the *M. lignano* gene annotation significantly expands the gene set, primarily by annotating additional paralogs in duplicated regions. Functional annotation of the obtained gene models was carried out using the EnTAP v2.1.0 pipeline based on homology in the Uniprot: UniRef90 proteins database accessed on 5 February 2025 (https://www.uniprot.org/help/uniref) and the output of the InterProScan v.5.61-93.0 program. Clustering of highly homologous paralogs was performed using CD-HIT v4.8.1with additional post-processing of clusters to collapse gene overlaps.

### 2.4. DEG Analysis

The updated gene annotation was used for the identification of differentially expressed genes (DEGs) between groups of intact worms from the DV1_8 and DV1_10 sublines, characterized by different numbers of copies of large chromosome 1 (two and four copies, respectively).

We previously detected that the reference genome assembly Mlig_3_7 contains quite a lot of copy number errors and chimeric sequences, mainly collapsed haplovariants, associated with challenges in resolving highly homologous regions in the three subgenomes L_1_, L_2_, and S. Accordingly, the assessment of gene copy number using the existing version of the reference genome assembly of *M. lignano*, Mlig_3_7, turns out to be not entirely correct. In this case, the solution is to simultaneously sequence the genome (WGS) and transcriptome (RNA-seq) of each sample, which will allow the copy number of a gene to be taken into account when assessing its differential expression. WGS data from [21] were aligned to the reference genome using bowtie2 v2.4.4. Tables of read counts were produced with featureCounts command of the Subread package with the following parameters: “-M -Q 0 –O –p --countReadPairs –t gene” for WGS data and “-M –Q 0 –s 2 –p –countReadPairs –t exon” for RNA-seq data. The exonic content of genes was counted for RNA-seq libraries, and the whole gene body was counted for low-pass WGS data; if the gene was shorter than 10 Kb, it was expanded in both directions to this minimal length. Further processing and statistical analysis of differential gene expression was performed in the R environment with Genomic Ranges and DESeq2 packages. Additionally, the ratio of WGS coverage (cr) between the DV1_8 (SSL_1_L_2_) and DV1_10 (SSL_1_L_1_L_2_L_2_) sublines was used to assign the detected genes to the subgenomes S and L_1_/L_2_ using fixed thresholds: cr >= 1.375 for S and cr <= 1.125 for L_1_/L_2_.

For the subgenome S, the expected coverage ratio is 1.5, while for L_1_/L_2_, the expected ratio is 1.0. Due to the possible biological variation and sequencing errors, we did not use the exact theoretical values. Instead, we set buffer zones to ensure high confidence: cr >= 1.375 for subgenome S, because this value is the midpoint between the theoretical expectation for S, cr = 1.5, and the expected ratio for L, cr = 1.25. By setting the bar at 1.375, we could be confident that any gene with a coverage ratio closer to 1.5 than to 1.25 is statistically likely to belong to the subgenome S. For L_1_/L_2_, the threshold 1.125 served as the lower bound to filter out genes that show a balanced dosage. It ensures that only genes consistently appearing in the expected 1:1 or 2:2 ratio across both sublines are assigned to the subgenomes L_1_/L_2_, filtering out potential noise that might lean toward S. By leaving a gap between 1.125 and 1.375, we created a so-called “no man’s land,” and, for genes falling in this middle range, we considered them ambiguous and excluded them from the assignment.

WGS data were used to create a gene-level matrix of size factors, which was further incorporated together with RNA-seq size factors in the general linear model of RNA-seq data to account for variations stemming from different copy numbers of genes in two sublines or copy number errors in the reference genome. Briefly, WGS counts were initially normalized on both the sequencing depth (DESeq2′s median of ratios method) and gene length for each subline independently. The resulting counts with bimodal distribution were used to extract the subset of genes belonging to subgenomes L_1_/L_2_ based on a 1.5-fold difference of coverage between the groups. The matrix of WGS counts was again depth-normalized based on the above subset of genes and scaled. In cases of low gene coverage by the WGS library, we imputed the coefficient from its nearest gene. Finally, the default calculated vector of RNA-seq size factors was multiplied by this matrix and used to construct the general linear model implemented in the DESeq2 package [35]. Genes having mean RNA-seq counts less than 10 in both groups were excluded from further analysis. The constructed model allowed us to detect changes in gene expression that are not linearly related to changes in gene copy number, including the effects of gene dosage compensation.

### 2.5. Morphometry

The measurements of the morphometric traits of worms were conducted, as described earlier [10]. We measured adult worms (*n* = 24) from each subline. Measurements for each worm included the total body length and width, body area, sizes of gonads (including gonads’ area), eyes (diameter), stylet (proximal and distal openings, straight and fragmented length), and sperm (feeler, body, bristle, shaft, and brush). The detailed morphology of sperm of *M. lignano* can be found in Figure S5 [10,29]. The morphometry of mature sperm (*n* = 54 for each subline’s worms) was carried out according to the protocol [29].

### 2.6. EdU Staining

The dividing cells were stained by adding in the EdU (5-Ethynyl-2-deoxyuridine, 20 μM) staining solution (Lumiprobe, Moscow, Russia) to the culture medium containing worms of different ages, i.e., hatchlings (h) and adult mature worms (a). The EdU-labelled cells were detected using a click reaction with Cyanine3 azide in the presence of a copper catalyst, according to the manufacturer’s protocol (Lumiprobe, Russia). Additionally, the number of EdU-labelled cells (i.e., S-phase cells) in the testes of adult worms can serve as a dynamic measure of testicular activity [36]. We assumed the number of EdU-positive cells as the proliferative activity of cells in gonads (testes and ovaries). The number of EdU-positive cells in adults was counted using cellSens software (latest v. 4.2). Fluorescent specific signals from EdU-stained cells were counted for hatchlings (*n* = 30 per each subline) and gonads (testes and ovaries separately) of adult worms (*n* = 30 per each subline) derived from the DV1_8 and DV1_10 sublines.

### 2.7. Microscopy Analysis

Morphometric measurements were conducted on live specimens of *M. lignano* using an AxioImager.A2 microscope (Zeiss, Oberkochen, Germany) with transmitted light. The microimages and measurements were obtained using the software ImageView version x64 (Olympus, Tokyo, Japan).

Microscopic analysis of EdU-labeled cells was performed using the confocal laser-scanning microscope Olympus FW3000, using the software OLYMPUS FLUOVIEW FV31S-DT (Olympus). To count EdU-positive cells in hatchlings, the focus stacking or z-projection was applied (merging z-stacks into one plane, which creates a single composite microimage with an extended depth of field from multiple images taken at different focal planes).

### 2.8. Brood Size Measurements

Eu- and aneuploid worms from the DV1_8 and DV1_10 sublines were synchronized via egg laying. Then, we harvested hatchlings and isolated them separately in 24-well plates with f/2 medium containing diatom algae to allow the hatchlings to develop into adult worms. After maturation, the worms were plated together as virgins and were held in large mating groups of 80 in Petri dishes (one mating group for each subline) for one day. To measure the brood size and identify potential reproductive defects associated with ploidy differences, worms were subsequently isolated into individual wells of 24-well plates. These individuals were transferred to fresh plates every 2–3 days. In total, 80 DV1_8 and 78 DV1_10 fertilized worms were monitored over a 14-day period. Eggs were counted and checked daily for hatching; the time from laying to hatching was recorded to assess the developmental time (from egg laying to hatching) of offspring in both sublines and then counted for viability (hatched vs. unhatched eggs). The hatchlings were isolated in 24-well plates with f/2 medium for two weeks to allow them to grow. Then, we fixed the number of mature and malformed (no growth, lack of or incomplete formation of gonads, etc.) worms in the progeny from the DV1_8 and DV1_10 sublines.

### 2.9. Statistical Analysis

Quantitative data were tested for normality using the Shapiro–Wilk test, and homoscedasticity across the DV1_8 and DV1_10 sublines was assessed using Levene’s test. For datasets meeting the assumptions of normal distribution and equal variance, Student’s *t*-test was used to compare means. Non-normally distributed datasets (*p* < 0.05) were analyzed using the Mann–Whitney U test.

Kaplan–Meier survival analysis was employed to estimate the probability of offspring survival across developmental stages. The primary event of interest was defined as the mortality or the development of abnormal morphology. Survival probabilities were calculated at each stage (hatchling, juvenile, and adult), and the cumulative survival probability was estimated by multiplying these sequential probabilities. Differences in survival between the DV1_8 and DV1_10 progeny were statistically compared using the log-rank test. In all analyses, a *p*-value < 0.05 was considered statistically significant.

All statistical analyses were carried out using commercially available software (STATISTICA version 13 by StatSoft Inc., Tulsa, OK, USA).

### 2.10. Validation of RNA-Seq Datasets Using a Droplet Digital PCR (ddPCR)

Three biological replicates were established (75 worms for each replicate) for the DV1_8 and DV1_10 sublines. For verification of the RNA-seq analysis, we used three technical replicates per each biological replicate. To validate the WGS-informed normalization model across different genomic scenarios, we selected five genes for ddPCR analysis: one stable housekeeping control (COX5B), two genes identified as non-differentially expressed (non-DEGs: C14077 and C14804), which scale linearly with gene dosage, and two target differentially expressed genes (DEGs: 565G9 and 1804G1), which show significant deviations from the expected dosage effects. Primers were designed using Primer3Plus (http://www.bioinformatics.nl/cgi-bin/primer3plus/primer3plus.cgi, accessed on 5 February 2025). The primers and probes are listed in Table S1.

Total RNA was extracted using “Lira” reagent (Biolabmix, Novosibirsk, Russia), according to the manufacturer’s protocol. To prevent possible genomic DNA contamination, all specimens were treated with RNAse-free DNAse I based on the manufacturer’s recommendations (New England Biolabs, M0303S). Then, the mRNA was reverse transcribed using ProtoScript^®^ II First Strand cDNA Synthesis Kit (New England Biolabs, E6560S), according to the manufacturer’s instructions. The transcript abundance was measured using the Bio-Rad QX100 ddPCR System (Bio-Rad Laboratories, Inc., Hercules, CA, USA). The ddPCR SuperMix for probes reaction mixture (no dUTPs, Bio-Rad Laboratories, Inc., #1863023) was used for DEGs’ validation. Three technical replicates of the PCR mix for target and reference transcripts and a no-template control were included in each ddPCR run. In brief, each 22 μL 1x ddPCR SuperMix reaction mixture containing cDNA templates and forward and reverse primers (and two probes for the target and reference genes, respectively) with optimized concentration was mixed with 70 μL of Droplet Generation oil for Probes (Bio-Rad Laboratories, Inc., # 1863005) in a DG8 Cartridge (Bio-Rad Laboratories, Inc., USA, # 1864008). The probes were 5′-labeled with 6-carboxyfluorescein (FAM) or 6-carboxy-2, 4, 4,5, 7, 7 hexachlorofluorescein succinimidyl ester (HEX) as the reporter and BHQ-1 as the 3′-labeled double quenchers (Biolabmix, Novosibirsk, Russia). The cartridge was covered with a DG8 gasket and loaded into the QX100 Droplet Generator (Bio-Rad Laboratories, Inc.) to generate PCR droplets. From each droplet mix, 40 μL was transferred to a 96-well PCR plate (Bio-Rad Laboratories, Inc., # 12001925) and sealed using PX1™ PCR plate Sealer (Bio-Rad Laboratories, Inc.). PCR thermal cycling was optimized, and the amplification signals were read using the QX100™ Droplet Reader and analyzed using QuantaSoft software v1.7.4.0917 (Bio-Rad Laboratories, Inc.).

The log_2_ fold change (log_2_FC) of each examined target transcript (DV1_10 vs. DV1_8) worm was calculated after normalization to the reference genes (COX5B, C14077, and C14804). Taking into account the belonging of 656G9 to the subgenome S (that means its copy number in the DV1_10 is the same as in the DV1_8) and 1804G1 to the subgenome L_1_ or L_2_, correspondent correction was conducted on the raw ddPCR data. To accomplish this, we multiplied the value of the target transcript 656G9 (copies/μL) by 1.5 times and transcript 1804G1 (copies/μL) by 0.75 times in the DV1_10 replicates.

## 3. Results

In comparison to euploids, the genome of an aneuploid worm of *M. lignano* with two additional L chromosomes is about 1.5 times larger than that in euploids. In many organisms, the increased ploidy results in enlarged sizes at different levels, from cellular to organismal [37]. We measured the sizes of the body, gonads, the stylet, and sperm in euploid and aneuploid worms of two sublines (DV1_8, euploid worms, 2n = 8; SSL_1_L_2_ and DV_10, aneuploid worms, 2n = 10; SSL_1_L_1_L_2_L_2_). The increased sizes of all of them were revealed in aneuploid worms (Table 1 and Table S2). Earlier increased testes were observed in large mating groups in *M. lignano*. Probably, this enlargement was associated with increased cellular activity [36]. The worms of the sublines DV1_8 and DV1_10 are kept in our laboratory in mating groups of the same size; therefore, the increased gonads’ size could not be linked to the mating-group size but could be associated with increased cell proliferation in testes of worms with increased genome size.

The sperm of *M. lignano* has special morphological traits (Figure S5), including a feeler, a sperm body, a pair of lateral bristles anchored at the junction of the sperm body and the shaft, the shaft containing the nucleus, and a brush posterior to the shaft [38]. Sperm morphology and size are important factors for sperm competition. The feeler anchors sperm to the epithelium of the antrum, while lateral bristles have been predicted to reduce the likelihood of sperm being removed from the antrum during sucking behavior post copulation [36,38]. Measurements of the main sperm traits revealed that the worms with the 2n = 10 karyotype produced larger sperm (Table 2 and Table S2), having longer bristles and feelers. The longer bristles help sperm to avoid being sucked out [39], and longer feelers possibly allow better positioning in the antrum [40]; this increases the chance of successful fertilization of an egg.

### 3.1. Cell Proliferative Activity in Euploid and Aneuploid Worms

**Figure 2:**
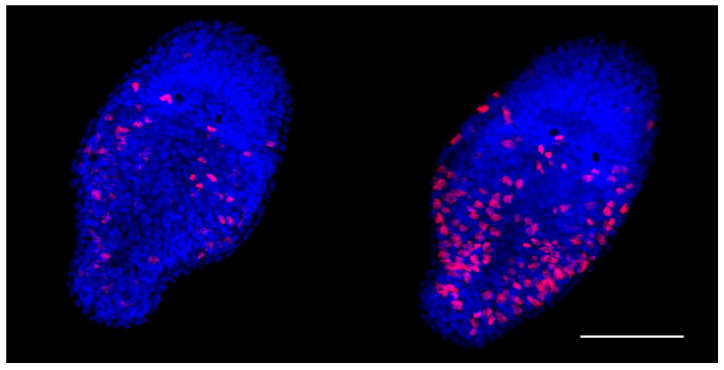
Cells involved in proliferation in the one-day-old euploid (**left**) and aneuploid (**right**) hatchlings detected with the EdU proliferation assay. EdU-labeled cells are in red. Scale bar 50 µm.

The EdU proliferation assay is a sensitive and reliable technique for detection of the cell proliferation. A one-day-old *M. lignano* hatchling consists of about 1500 cells; fifty of these are neoblasts [41]. In our study, the average number of cells involved in proliferation in one-day-old euploid hatchlings appeared to be 71.03 ± 19.79. Unexpectedly, in the aneuploid hatchlings, the registered proliferative activity of the cells was significantly higher (Figure 2, Table 3). The cells with the EdU-positively stained nuclei were distributed throughout the body of aneuploid hatchlings, excluding their head region. There were only a few nuclei found in the head region of both eu- and aneuploid worms (Figure 2).

**Figure 3:**
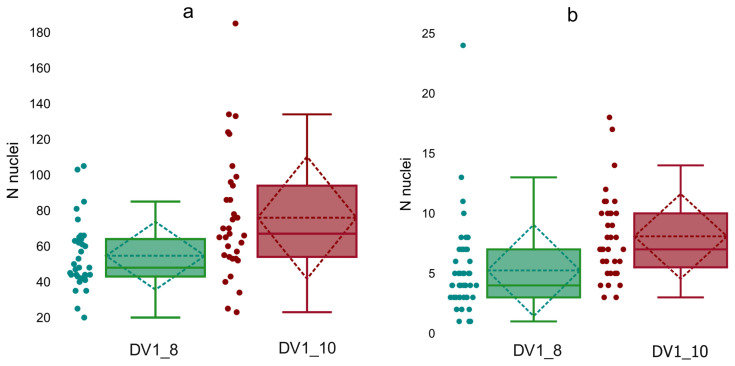
Distribution of EdU-labeled nuclei in testes (**a**) and ovaries (**b**) of adult worms from the DV1_8 (green color) and DV1_10 (red color) sublines.

We labelled most of the EdU-labeled cells as neoblasts and proliferating differentiating cells. Besides regeneration of the damaged and lost tissue or organs in *M. lignano*, neoblasts also renew them. In adults, about 160 neoblasts [41] occur, evenly distributed throughout upper ‘layers’ of the body and in bands located laterally [42]. However, we revealed cells with the EdU-labeled nuclei throughout the worm body, including various tissues and organs. In contrast to the hatchlings, the large number of the EdU-labeled nuclei made their count difficult. We have counted EdU-positively stained nuclei only in the testes and ovaries of the euploid and aneuploid worms. Note, in *M. lignano*, the testes’ periphery contains spermatogonia and primary and secondary spermatocytes [43], while the maturing spermatids and mature sperm are located in the inner region of the testes. The anterior part of the testes is enriched for proliferating cells and serves as a ‘proliferative center’ [44]. The ovaries mainly consist of oogonia and oocytes. In both, eu-and aneuploids, the primordial germ cells, progenitors of sperm and ova, are located at the periphery of testes and ovaries, respectively. In the examined specimens of worms, the testes and ovaries of the DV1_10 worms in comparison with the DV1_8 worms contained more cells with labeled nuclei (Table 3, Figure 3).

### 3.2. Brood Size Measurements of Worms from the DV1_8 and DV1_10 Sublines

The virgin adult worms from the DV1_8 and DV1_10 sublines were kept for one day in two large mating groups of the same size (*n* = 80). Then, all worms were isolated for the estimation of the egg laying and successful brood size in the DV1_8 and DV1_10 sublines. The number of laid eggs, hatchlings, alive worms, and worms of normal size and morphology produced by 80 DV1_8 and 78 DV1_10 worms were counted (Table 4). Six DV1_8 and eight DV1_10 worms laid no eggs, while 17 DV1_8 and 26 DV1_10 worms laid no hatched eggs. At the hatchling stage, the specimens were isolated in wells with algae. Further observations showed that they, being virgin, appeared to be capable of laying eggs. Since *M. lignano* is an obligate cross-fertilized worm, we referred to these eggs as unfertilized. Therefore, in our experiments, part of the counted unhatched eggs could also be unfertilized. The number of laid eggs had sharply decreased by the sixth day. However, later the worms began laying eggs again, with varying frequency and in varying quantities (Figure S6), but none of them hatched. Probably, most or all of them were unfertilized. Supposing that eggs laid after the sixth day were mostly unfertilized ones, we consider the eggs laid for the first six days of the experiment separately (Table 4). However, some unfertilized eggs could be laid also on the first day of the experiment. Therefore, even for the eggs laid in the first six days, we cannot be sure that all of them were fertilized ones. Due to this problem, the data on the fertilized egg number produced by the DV1_8 and DV1_10 worms were difficult to interpret and analyze.

For this reason, special attention was paid to the hatching and further development of the specimens. We should note that, normally, worms lay about one single-cell fertilized egg (zygote) per day [14], and the time between the egg laying and hatching is around five days [18]. In our experiment, egg hatching took place mainly three days after egg-laying in both euploid and aneuploid worms. In rare cases, egg hatching was observed later, e.g., in five, six, or even 10 days. All of these cases were registered for the worms of the DV1_8 subline (Figure S7).

**Figure 4:**
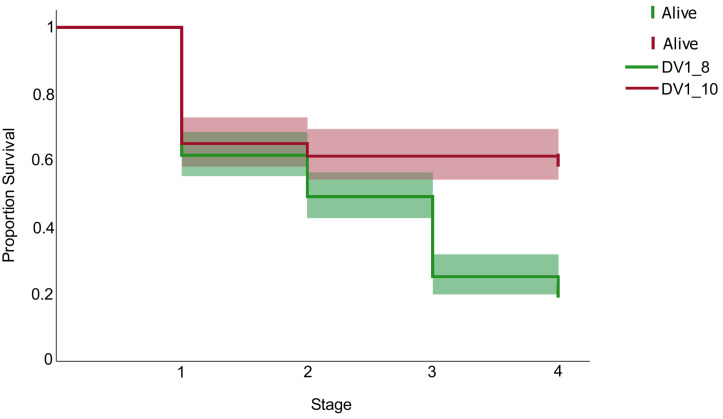
Kaplan Meier analysis for estimation of offspring survival in the DV1_8 (red) and DV1_10 (green) sublines. The X-axis represents developmental stages (1: egg; 2: hatchling, 3: juvenile; 4: adult worm).

Any disorders in the worm’s ontogenesis at any development stage negatively affect the worm’s maturation and normal phenotype formation. We tracked the survival of individuals across all developmental stages, from egg laying to maturity. In a Log-Rank test, a chi-square of 42.62 and a *p*-value < 0.001 indicate a highly statistically significant difference between the survival curves of the compared sublines DV1_8 and DV1_10. In the DV1_8 subline, mortality occurred continuously at all life stages, including unhatched eggs, hatchlings, and juveniles. Remarkably, only 21.05% of eggs laid by the DV1_8 worms successfully produced adults with a normal phenotype (standard body size and fully developed organ systems) (Figure 4, Table 4). Most DV1_8 hatchlings exhibited stunting and failed to reach normal body sizes; these individuals, along with abnormally developing juveniles, typically died shortly after reaching their respective stages (Figure 4).

In contrast, the DV1_10 subline exhibited a distinct survival pattern. While significant mortality occurred primarily at the egg stage (unhatched eggs), those individuals that successfully hatched demonstrated remarkably high viability. For most DV1_10 replicates, the number of mature worms in offspring coincided almost exactly with the number of hatched eggs (Figure 4 and Figure S8), resulting in a significantly higher overall production of normal adult worms (60.25%) compared to the DV1_8 worms (Figure 4, Table 4).

### 3.3. Aneuploidy-Driven Changes in the Transcriptome of M. lignano

For comprehensive genome-based analysis of the transcriptomes of the two sublines, we modified the original Mlig_3_7 reference gene annotation using multi-match mapping of both original Mlig_3_7 genes and transcripts assembled from the newly obtained RNA-seq data. The resulting consensus annotation significantly expanded the list of detected duplicated genes.

**Figure 5:**
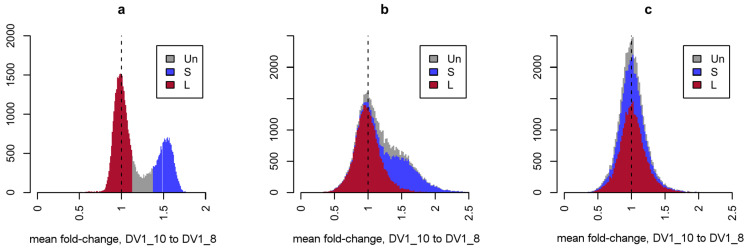
Distribution of inter-strain fold-changes obtained for WGS and RNA-seq data with different normalizations. Values for depth-normalized WGS (**a**) and RNA-seq (**b**) data and RNA-seq data that have been additionally normalized to gene dosage (**c**). The color indicates the predicted assignment of the corresponding genes to the L_1/_L_2_ (red) or S (blue) subgenomes or unassigned genes (grey), based on the fixed fold-change threshold for WGS data.

Direct comparison of RNA-seq data for the two sublines, normalized for sequencing depth, revealed a global increase in the expression of a large number of genes in aneuploid worms (Figure 5). Since we previously showed that the copy number of the three subgenomes within the genomes of these lines differs (SSL_1_L_2_ vs. SSL_1_L_1_L_2_L_2_), it was logical to assume that the observed differences in the expression of paralogous genes are due to differences in their dosage. To normalize transcriptome data for gene dosage in the genome, we used previously obtained WGS data for the worms of the DV1_8 (euploid) and DV1_10 (aneuploid) sublines [21]. This operation also eliminates any discrepancies in gene copy numbers between the reference and studied genomes and allows us to label annotated genes as belonging to the subgenomes S or L_1_/L_2_ (Figure 5a). Normalization for gene dosage eliminated the pattern of global differences between the transcriptomes of euploid and aneuploid worms, which affected genes on small chromosomes (Figure 5b,c). Thus, under conditions of variable ploidy level and subgenome composition, the expression of the vast majority of the *M. lignano* genes is directly proportional to their genome dosage.

According to the results of the RNA-seq analysis, the vast majority of genes are expressed in proportion to their copy numbers in the genome of *M. lignano*. In spite of this, changes in the expression level for some genes are not correlated with differences in their copy numbers.

**Figure 6:**
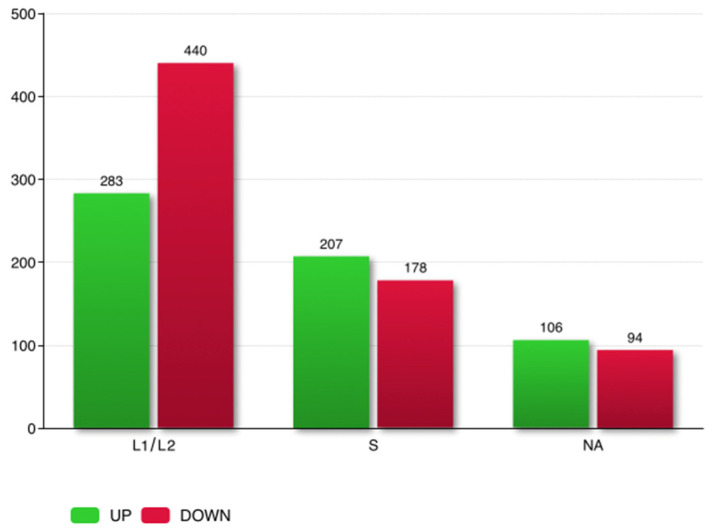
DEGs belonging to the subgenomes L_1_/L_2_ and S and DEGs with unknown attachment (NA). Up-and downregulated genes are indicated by colors (green and red, respectively). The numbers of genes are on the top of each bar.

In total, 1776 genes out of 52,294 analyzed genes were defined as differentially expressed (adjusted *p*-value < 0.05) between the DV1_8 and DV1_10 worms. Based on absolute FC >  1.5, DEGs were screened. In total, 1308 genes were differentially expressed between worms of the DV1_8 and DV1_10 sublines (Table S4a). Overall, in aneuploid worms 596 genes were upregulated, while 712 were downregulated, compared with their level of expression in euploid worms. Based on the ratio S/L using the WGS data, we were able to predict the belonging of most DEGs to the subgenome S or subgenomes L_1_ and L_2_ taken together. Most of the DEGs were assigned to the L subgenomes (723 genes), while 385 genes were assigned to the subgenome S (Figure 6, Table 5 and Table S5). This indicated that aneuploidy on the large chromosome affects the expression of genes encoded by all three subgenomes, including genes from the small chromosomes (subgenome S).

To demonstrate the robustness of these assignments, a sensitivity analysis was performed by systematically varying the coverage ratio thresholds (Table S4b). We tested a range of threshold combinations, from highly stringent (e.g., cr ≥ 1.5) to more permissive (e.g., cr ≥ 1.30). The analysis revealed that the number of assigned DEGs remained highly stable across all tested parameters. Specifically, the overall proportion of DEGs assigned to S versus L_1_/L_2_ did not change significantly. Some DEGs with intermediate coverage ratio values that fell within the “no man’s land” gap (Figure 5a, grey color) were categorized as NA (200 genes with our primary threshold; Figure 6). However, the consistency of the results across the full range of thresholds confirms that our approach is robust and that the biological conclusions are not dependent on the specific thresholds selected.

The validation of expression levels for selected DEGs was carried out by qRT-PCR. The results showed a high consistency between the RNA-Seq and ddPCR data, confirming the high reliability of RNA-Seq. Both the constant expression of genes that served as intrinsic reference genes in the current study and the differential gene expression for a few target genes were validated for RNA-seq data using ddPCR (Figure S9).

### 3.4. Functional Enrichment Analysis for DEGs

The patterns of gene expression can be evaluated in a variety of ways, for example, by grouping genes based on pathway or function in GO or KEGG pathway analyses to identify which pathways are up- or downregulated between groups.

**Figure 7:**
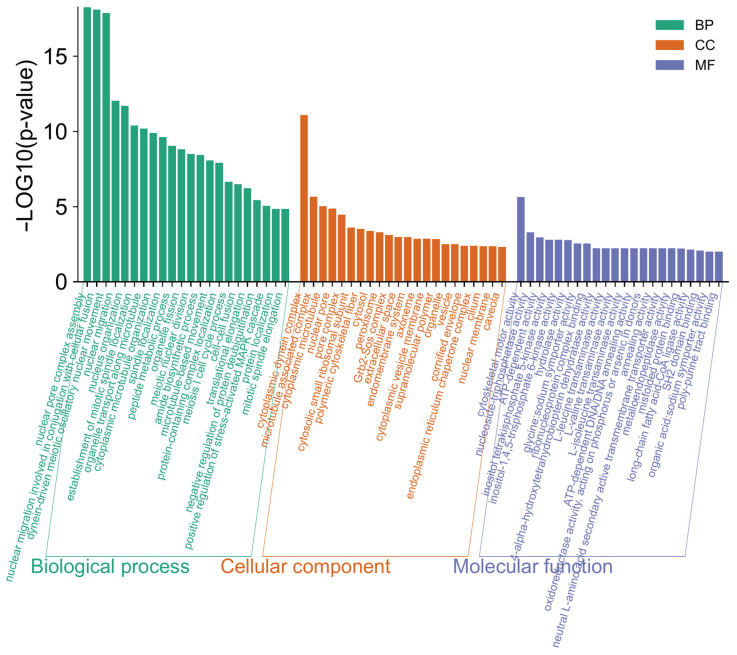
Distribution of 22 top DEGs into gene ontologies by GO classification of transcripts biological process (BP), cellular component (CC), and molecular function (MF).

Ploidy-specific transcriptional differences were further explored through an enrichment analysis of Gene Ontology (GO) terms. DEGs in euploids vs. aneuploids were annotated using the GO database and classified into three major GO categories, namely, biological process, cellular component, and molecular function, which were then further classified into functional subcategories (Tables S6 and S7). The DEGs were mostly enriched in GO terms linked with the primary GO category ‘Biological Process’, totaling 191 GO terms (Figure 7, Table S7).

In this category, ‘nuclear pore complex assembly’, ‘nuclear migration involved in conjugation with cellular fusion’, and ‘dynein-driven meiotic oscillatory nuclear movement’ were the most highly represented GO terms. In the cellular components, ‘cytoplasmic dynein complex’ was the most represented term. In the molecular functions category, ‘cytoskeletal motor activity’ was the dominant GO term. The predominant GO terms represent a general overview of content of gene ontologies and exhibit a wide range of active processes occurred in the *M. lignano* worms with increased genome ploidy.

To identify the biological processes involving the DEGs, we conducted a KEGG pathway analysis. In total, KEGG terms were assigned to 1308 differentially expressed genes, and only 354 DEGs were classified into five broad categories (Tables S8 and S9). The DEGs were distributed across ‘Organismal Systems’ (28.43%), ‘Metabolism’ (22.67%), ‘Environmental Information Processing’ (10.29%), ‘Cellular Processes’ (7.51%), and ‘Genetic Information Processing’ (7.38%).

KEGG enrichment analysis highlighted several pathways, though functional inferences for *M. lignano* should be treated with caution as these annotations are based on orthology to better-characterized vertebrate systems. Within the ‘Organismal Systems’ category, enriched pathways included the NOD-like receptor, GnRH, and PPAR signaling pathways, as well as vasopressin-regulated water reabsorption and the renin-angiotensin system. While flatworms lack the canonical vertebrate GnRH signaling architecture, orthologous signaling components have been described in invertebrates like *C. elegans*, where AKH/GnRH-like peptides are putatively involved in the modulation of egg-laying timing [45]. Similarly, the presence of ‘platytocin’ in some marine flatworms suggests that pathways annotated as ‘vasopressin-regulated’ (ko04962) may be orthologous to pathways that putatively participate in physiological adaptations to salinity fluctuations or reproductive organization [46]. These annotations serve purely as orthology-based indicators of potential conserved functions, rather than confirmed functional equivalents.

The ‘Metabolism’ category included pathways associated with lipid metabolism (e.g., fatty acid elongation, biosynthesis of unsaturated fatty acids) and carbohydrate metabolism. Within ‘Genetic Information Processing’, enriched pathways such as ubiquitin-mediated proteolysis and RNA degradation were identified. These may be linked to the maintenance of homeostasis through ubiquitin-mediated proteolysis via the ubiquitin-proteasome system, a major pathway for protein degradation, which regulates the amounts of many proteins for the maintenance of and the regulation of transcript abundance, which are particularly relevant in the context of increased genome ploidy.

Finally, the ‘Environmental Information Processing’ category included the mTOR signaling pathway (ko04150). In other flatworms, such as planarians, mTOR signaling has been implicated in the regulation of neoblast proliferation during cell turnover and regeneration. While the enrichment of this pathway in our dataset is hypothesized to correlate with the observed proliferative states in *M. lignano*, we emphasize that substantial functional validation remains required to establish a definitive link between these transcriptional patterns and specific phenotypes such as increased gonads’ size or stem cell activity.

### 3.5. Tissue-Specific DEGs Linked with Changes in Ploidy Level

GO or KEGG enrichment analysis was unable to assign the term for many genes of *M. lignano*, including differentially expressed genes, at least in part due to the prominent gene novelty in *M. lignano*. As shown earlier [44], out of 2604 germline-enriched transcripts, only 979 (37.60%) had homologs in human and 1067 (40.98%) in *S. mediterranea*, while 1412 (54.22%) were predicted to be *M. lignano*-specific. In this situation, an alternative source of functional information on genes with broad coverage is the results of other genome-wide studies of *M. lignano* and in particular data on tissue-specific gene expression.

To define the tissue or organ system enriched with DEGs, we used the transcriptome data from published studies on *M. lignano* [20,44,47] and linked with databases.

**Figure 8:**
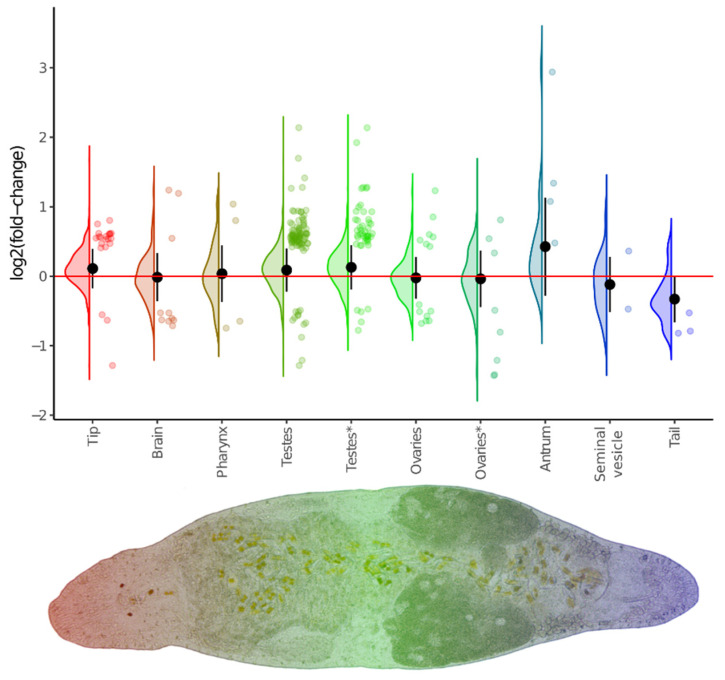
Violin/jitter plots for differences between euploid and aneuploid worms in expression of region-specific genes. Classification of region-specific gene sets is taken from the database linked with [44]. Additionally, ovaries- and testes-specific genes were refined from [47] and marked by an asterisk. Violin plots illustrate log_2_FC distribution for the whole set of detected genes, while jitter plots show only differentially expressed genes. Black circles and whiskers show the mean and standard deviation of the violin plot.

We found that a substantial number of DEGs exhibited spatially specific expression (Figure 8). Perhaps the most prominent of these were the tip- and testes-specific genes, whose expression was increased in the DV1_10 worms. Furthermore, the expression of the entire set of these region-specific genes was also elevated. The latter likely indicates an increased proportion of the corresponding organs in the body of the DV1_10 worms. This finding is strikingly consistent with the observed the increased sizes of gonads and more active proliferation in the testes and ovaries; we took a closer look at the gonad-specific DEGs that may orchestrate these phenotypic changes. In total, we defined 33 gonad-specific DEGs; of these, 28 had specific expression in testes (twenty-six up- and only two downregulated genes), and five genes had ovary-specific expression (one up- and four downregulated). The upregulated testes-specific DEGs were associated with the Hedgehog signaling pathway (ko04340), the Hippo signaling pathway (ko04390 and ko04391), and the Wnt signaling pathway (ko04310) (Table S10).

## 4. Discussion

The unique genome organization of *M. lignano* allowed us to establish a neopolyploid model to study the genomic and phenotypic consequences of ploidy changes. The DV1_8 worms represent hidden tetraploids (SSL_1_L_2_), while the DV1_10 worms are hidden hexaploids (SSL_1_L_1_L_2_L_2_) due to the tetrasomy of the large chromosome 1. Consequently, the genome of the DV1_10 worms is 1.5 times larger than that of the DV1_8 worms.

In our study, we revealed the distinct survival patterns between the two sublines. In the DV1_8, mortality occurred continuously across all developmental stages, with only 21.05% of eggs developing mature worms. Because the SSL_1_L_2_ genotype should produce SSL_1_L_1_ and SSL_2_L_2_ offspring, the observed developmental disorders and stunting likely result from the segregation of these homozygous genotypes. The independent evolution of subgenomes L_1_ and L_2_ through subfunctionalization or gene loss possibly mean that homozygosity likely reveals deleterious recessive alleles or lethal gene dosage imbalances, leading to elimination at various stages of ontogenesis. This allows us to suggest that all mature DV1_8 worms used in our morphometric and transcriptomic analyses possessed the heterozygous SSL_1_L_2_ genome.

In contrast, the DV1_10 subline exhibited a selection bottleneck at the egg stage. While massive mortality occurred primarily among unhatched eggs, the individuals that successfully hatched demonstrated remarkably high viability, with 60.25% of total eggs producing normal adults. In many specimens, the number of mature offspring coincided almost exactly with the number of hatched eggs. This high post-hatching stability suggests that the hexaploid state in DV1_10 acts as a constitutive buffer that mitigates the segregation of lethal alleles, whereas the DV1_8 subline is dependent on stringent post-zygotic selection to maintain genomic integrity. Once the initial hurdle of embryonic development has been overcome, the increased ploidy may mask deleterious variants more effectively, facilitating the evolutionary success of the hexaploid DV1_10 worms. The distinct survival trajectories suggest that these sublines navigate different evolutionary landscapes. In DV1_8, the necessity for heterozygosity at the SSL_1_L_2_ locus creates a narrow ‘viability corridor,’ resulting in continuous attrition. While this introduces a survivorship bias toward heterozygous adults, it highlights the fragility of the DV1_8 genomic configuration. Conversely, the selection bottleneck in DV1_10 occurs almost exclusively during embryogenesis. The subsequent high post-hatching stability indicates that hexaploidy provides a more robust genomic buffer, effectively masking deleterious variants that would otherwise be eliminated by the continuous selection observed in DV1_8.

The observed phenotypic differences are reflected in our transcriptomic data. Comparison of transcriptomic patterns of the euploid and aneuploid worms revealed differences in their gene expression levels. Under ploidy changes and subgenome composition, the expression of the vast majority of the *M. lignano* genes is proportional to their copy numbers in the genome of *M. lignano*. In spite of this, 1308 genes were defined as differentially expressed between the euploid (SSL_1_L_2_) and aneuploid (SSL_1_L_1_L_2_L_2_) worms.

Overall, most of the DEGs were assigned to the L_1_/L_2_ subgenomes (723 genes), while 385 genes were assigned to the subgenome S. This indicated that aneuploidy on the large chromosome affects the expression of genes encoded by all three subgenomes, including genes from the small chromosomes (subgenome S). This aneuploidy-induced transcriptional response occurs [48], when genes with differential expression are not limited to the genes of aneuploid chromosome, the large chromosome of *M. lignano*, but include a significant number from euploid small chromosomes. Our findings in *M. lignano* align with established patterns in other eukaryotes: yeast, mice, and humans [49]. In yeast, the trans-effect of aneuploidy affects about 5–7% of genes, often manifesting as an Environmental Stress Response (ESR) that modulates genes involved in protein synthesis and folding, regardless of which chromosome is duplicated. In humans, clinical aneuploidies demonstrate a high proportion of trans-acting DEGs. For example, in fibroblasts with trisomy 21, nearly 88% of DEGs were distributed across other chromosomes [50]. Similarly, in sex chromosome aneuploidies like Turner (45,X) and Klinefelter (47,XXY) syndromes, over 75% of DEGs were identified in autosomes, while in carriers of karyotypes 47,XXX and 47,XYY, less than 30% of DEGs were autosomal [51]. The extent to which trans-effects of aneuploidy occur varies among species, and the underlying mechanisms are still poorly understood [52].

Thus, the phenotypic effects of aneuploidy may be associated either directly with changes in the copy number of genes located on the aneuploid chromosome or indirectly with changes in the expression of many genes on euploid chromosomes [49]. The result may be additive or synergistic expression and functional effects at the transcriptional and/or posttranscriptional levels [53]. This is consistent with the nonlinear nature of gene dosage effects that determine subsequent biochemical processes in the cell [54,55]. Although many of the biological effects caused by aneuploidy are consistent with the gene dosage balance hypothesis [56,57], it is worth considering the impact of the presence of extra chromosomes on the spatial nucleus organization and potentially on the genome-wide transcriptional activity of a wide variety of genes.

It should be noted that our transcriptomic analysis utilized three pooled biological replicates per subline. While this approach provided the necessary biomass for high-depth sequencing and allowed us to identify robust constitutive expression differences between the DV1_8 and DV1_10 sublines, it inherently masks individual-level transcriptomic heterogeneity. In aneuploid organisms, such heterogeneity—often referred to as phenotypic ‘noise’—can be significant [52]. However, our primary objective was to characterize the divergent evolutionary signatures and stable genomic buffering capacities that define these sublines as distinct populations. By pooling individuals, we effectively filtered out stochastic fluctuations, revealing the dominant transcriptional response associated with the change from a hidden tetraploid to a hexaploid state. Future studies employing single-worm RNA-seq will be instrumental in determining the degree to which individual aneuploid variation contributes to the broader patterns of selection and survival observed here.

Summarizing the list of revealed DEGs, many unfortunately remained outside functional annotation due to a lack of detectable homologs in other species. This observation indicates the presence of substantial gene novelty and suggests specificity within the *M. lignano*-lineage. Nevertheless, functional insights were gained from analyzing DEGs with significant changes in transcriptional activity.

One of the pathways identified is the GnRH signaling pathway, components of which are conserved across Metazoa. In *C. elegans*, this pathway is putatively involved in influencing egg-laying timing and progeny numbers [46]. Furthermore, functional annotation of upregulated testes-specific DEGs in the DV1_10 worms pointed toward an association with key signaling mechanisms, such as the Hippo, Wnt, and Hedgehog (Hh) pathways. These pathways have established roles in regulating cell proliferation, survival, and differentiation during spermatogenesis in model organisms. For example, the Hh pathway is considered crucial for normal testes development, and its disruption is known to lead to sperm defects and infertility in vertebrates [58]. Similarly, Wnt/β-catenin signaling plays essential roles in regeneration and development across various species, and in *M. lignano*, the Hippo pathway has been shown to regulate neoblast activity during development and regeneration [59,60].

Among the significantly enriched pathways for DEGs in the DV1_10 worms, special attention was paid to the mTOR pathway. This pathway plays key roles in the regulation of multiple fundamental processes that influence cell cycle, cell proliferation, organism growth, and survival in general biological contexts [61]. Ploidy changes can be associated with disorders in cell division such as asynchronous cell cycles, increased frequency of endomitosis, and genomic instability. The mTOR signaling pathway participates in controlling translational initiation and G1 progression. While these connections are well-documented in model systems, the specific functional consequences in *M. lignano* require further study.

Another significantly enriched pathway was linked with ubiquitin-mediated proteolysis and RNA degradation. These mechanisms are crucial for maintaining proteome homeostasis. The enrichment of these pathways may be associated with the challenges of maintaining balance under increased genome ploidy levels in the DV1_10 worms. Studies in other aneuploid organisms (e.g., yeast) have shown increased ubiquitination levels for proteins encoded on aneuploid chromosomes [62], potentially suggesting a role for the ubiquitin–proteasome system in buffering gene dosage imbalances [63].

It is worth noting the enriched metabolic pathways in the DV1_10 of *M. lignano*, especially carbohydrate and lipid metabolism pathways. These findings tentatively suggest that the DV_10 worms might exhibit advantages in carbon metabolism that could hypothetically correlate with the larger body sizes observed. It is known that various metabolic pathways regulate somatic and germline stem cell behavior. In some marine flatworms, ‘platytocin’ (a neuropeptide derived from the vasopressin/oxytocin-type gene) has been suggested to function as an ‘antidiuretic hormone’ and may be involved in organizing reproduction and chemosensory behavior [46]. We propose that genes involved in these processes may improve physiological adaptations to new environments by regulating body fluid chemical content under salinity fluctuations, though this interpretation is based on homology and requires functional testing for validation within this specific system.

## 5. Conclusions

In this study, we exploited the unique subgenomic organization of *M. lignano* to propose a new neopolyploid animal model for studying the transcriptomic and phenotypic traits under ploidy changes. We revealed that while the majority of the transcriptome maintains gene dosage balance proportionality relative to genome ploidy, 1308 genes exhibit a distinct aneuploidy-induced transcriptional response. The distribution of DEGs across both the large (L_1_/L_2_) and small (S) chromosomes highlights the presence of trans-effects, when aneuploidy on a single chromosome triggers shifts in expression of genes throughout the entire genome.

Our findings reveal a possible association between increased ploidy and important biological processes, linked to cell cycle regulation (mTOR signaling), proteome homeostasis (ubiquitin-mediated proteolysis), and metabolic adaptation. Furthermore, the correlation between specific signaling pathways, such as Hippo, Wnt, and Hedgehog, and the observed phenotypes provides a foundation for understanding the biological significance of aneuploid forms in this species. Further functional validation remains necessary to confirm definitive causal relationships between specific patterns of differentially expressed genes and the observed phenotypes.

Ultimately, our findings regarding the transcriptomic differences between the DV1_8 and DV1_10 sublines suggest that aneuploidy-driven ploidy changes induced complex effects on gene expression. The interactions between these DEGs play essential roles in the homeostasis, fecundity, and viability of the *M. lignano* aneuploids. These results suggest that *M. lignano* has evolved robust mechanisms to buffer the deleterious effects of aneuploidy, a trait that may contribute to its remarkable genomic plasticity. These features make *M. lignano* an attractive model for studies of the early stages of reorganization and functioning at the genome, transcriptome, and proteome levels in animal neopolyploids.

## Figures and Tables

**Table 1:** Morphometry of the DV1_8 and DV1_10 adult worms.

Subline	Body			Eye	Testis		Ovary		Stylet			Segmented L
	L	W	A	d	L	A	L	A	Distal Opening	Proximal Opening	Straight L	
DV1_8	1255.16 ± 197.92	166.89 ± 15.87	508,642.71 ± 37,772.35	8.12 ± 0.56	109.73 ± 16.5	4147.32 ± 1112.47	54.74 ± 13.24	1510.19 ± 485.92	4.82 ± 0.66	14.58 ± 2.41	69.88 ± 3.91	70.39 ± 4.28
DV1_10	1480.83 ± 156.27 ***	183.44 ± 26.59 ***	520,780.83 ± 36,558.49 ***	8.39 ± 0.62 *	144.89 ± 16.61 ***	7272.15 ± 1331.13 ***	72.76 ± 23.89 **	2933.12 ± 1741.63 ***	4.76 ± 0.6	15.09 ± 2.46	75.68 ± 4.69 ***	74.39 ± 12.21 ***

The values represent means ± 1SD and include absolute length (L), width (W), diameter (d), or area (A). Significant difference is marked by asterisk(s) (*p* < 0.05) *, (*p* < 0.01) **, and (*p* < 0.001) ***.

**Table 2:** Morphometry of mature sperm.

Subline	*n*	Sperm				
		Total L	Body, L	Feeler, L	Brush, L	Bristle, L
DV1_8	54	53.85 ± 6.06	11.86 ± 1.50	17.01 ± 1.63	5.15 ± 0.86	11.79 ± 0.86
DV1_10	54	65.40 ± 3.06 ***	13.21 ± 1.06 ***	20.31 ± 1.48 ***	6.01 ± 0.51 ***	12.74 ± 0.76 ***

The values represent means ± 1SD and include absolute length (L) of different structures of sperm. Significant difference is marked by asterisks (*p* < 0.001) ***. Statistical analysis was conducted using Student’s *t*-test; *n* stands for the number of measured sperm.

**Table 3:** Number of EdU-labeled cells in the body in the one-day-old DV1_8 and DV1_10 hatchlings and in the gonads of the adult worms.

Subline	*n*	Mean ± SD	U	z	*p*-Value	r
hatchlings			56.50	−5.82	<0.001	0.75
DV1_8	30	71.03 ± 19.79				
DV1_10	30	102.33 ± 22.82 ***				
testes			315.50	−2.94	0.003	0.36
DV1_8	30	52.47 ± 16.48				
DV1_10	30	81.97 ± 31.24 **				
ovaries			369.50	−4.08	<0.001	0.46
DV1_8	30	5.22 ± 3.85				
DV1_10	30	8.11 ± 3.58 ***				

The means ± 1SD are shown. Significant difference is marked by asterisks (*p* < 0.01) **, and (*p* < 0.001) ***. Statistical analysis was conducted using Mann–Whitney U-test; *n* stands for the number of hatchlings, testes, and ovaries.

**Table 4:** Results of Kaplan–Meier survival analysis.

Subline	N Eggs	N of Dead Worms (N)	N of Alive Adult Worms	% of Alive Worms
DV1_8	209	165	44	21.05
DV1_10	161	64	97	60.25

**Table 5:** Top ten most differentially expressed up- and downregulated genes and whether they belong to the subgenomes L_1_/L_2_ or S.

Transcript ID	Log_2_FC	Adjusted *p*-Value	Subgenome
mikado.scaf1795G14	5.67	0.004391	L
mikado.scaf3150G9	5.49	0.003809	L
mikado.scaf656G9	4.26	1.92 × 10^−70^	S
mikado.scaf1804G1	3.59	9.03 × 10^−9^	L
mikado.scaf1592G1	3.16	0.022669	L
mikado.scaf1151G13	3.16	0.002523	L
mikado.scaf3332G6	2.99	1.8 × 10^−5^	S
mikado.scaf3157G7	2.89	0.001061	NA
mikado.scaf3771G4	2.88	0.024537	NA
mikado.scaf794G14	2.83	0.0418	L
mikado.scaf514G9	−2.29	0.00114	L
mikado.scaf1021G14	−2.32	0.007259	NA
mikado.scaf4546G2	−2.51	0.00025	L
mikado.scaf572G34	−2.55	0.009102	L
mikado.scaf528G22	−2.63	0.011372	L
mikado.scaf77G16	−3.16	1.31 × 10^−5^	S
mikado.scaf756G13	−3.31	0.002773	L
mikado.scaf1160G12	−3.33	5.58 × 10^−5^	L
mikado.scaf1116G34	−3.67	0.00151	L
mikado.scaf249G20	−4.93	0.01119	S

## Data Availability

The data presented in this study are available in the article and Supplementary Materials. The raw data supporting the conclusions of this article have been submitted to the NCBI BioProject database (https://www.ncbi.nlm.nih.gov/bioproject/, 5 February 2025) under accession number PRJNA951191.

## Supplementary Materials

The following supporting information can be downloaded at https://www.mdpi.com/article/10.3390/cells15030245/s1, Figure S1. Electropherograms for the RNA-seq libraries generated from the DV1_8 worm pools A, C, E; Figure S2. Electropherograms for the RNA-seq libraries generated from the DV1_10 worm pools A, C, E; Figure S3. Metrics for quality assessment of the generated RNA-seq libraries; Figure S4. Gene body coverage on average for each RNA-seq library; Figure S5. Transmission light microscopy of *M. lignano*; Figure S6. Dynamics of egg production and hatching during experiment; Figure S7. Development time from the egg to hatching for eu- and aneuploid worms; Figure S8. Fecundity of the euploid (DV1_8) and aneuploid (DV1_10) *M. lignano* worms; Figure S9. ddPCR validation for RNA-seq data using DEGs of the DV1_10 subline; Table S1. Design of primers and oligoprobes for ddPCR; Table S2. Statistical analysis of morphometric measurements of the DV1_8 and DV1_10 sublines; Table S3. Offspring production by the DV1_8 and DV_10 worms of *M. lignano*; Table S4a. Filtering out DEGs based adjusted *p*-value < 0.05; Table S4b. Sensitivity analysis of subgenome assignment thresholds; Table S5. Top ten most differentially expressed up- and downregulated genes and their belonging to the subgenomes; Table S6. DEGs functional classifications by GO (enriched sub-categories, *p*-value < 0.05), n: number; Table S7. GO terms enriched with DEGs into the category ‘Biological Process’; Table S8. KEGG pathway analysis for DEGs; Table S9. KEGG pathways for DEGs; Table S10. Testes- and ovaries-specific DEGs. Reference [64] is cited in Supplementary Materials.

## Abbreviations

The following abbreviations are used in this manuscript:

WGD	Whole-Genome Duplication
WGS	Whole-Genome Sequencing
ddPCR	Digital Droplet PCR
