# Supplementary material for: Mining Differentially Expressed Genes in the Marine Free-Living Flatworm Macrostomum lignano Under Aneuploidy-Driven Ploidy Changes
Source: Cells. 2026 Jan 27;15(3):245. doi: 10.3390/cells15030245 (PMC12896417; doi:10.3390/cells15030245)
Supplement: Supplementary file 1 [file cells-15-00245-s001.zip › Supplementary Materials (Figures S1-S9).pdf]

Supplementary Materials for

Mining differentially expressed genes in the marine free-living flatworm *Macrostomum lignano* under aneuploidy-driven ploidy changes

Kira S. Zadesenets [1,2,*] Nikita I. Ershov [1], Natalya P. Bondar [1,2], Konstantin E. Orishchenko[1,2], and Nikolay B. Rubtsov [1,2]

[1] Institute of Cytology and Genetics, Russian Academy of Sciences, Novosibirsk, Russian Federation Affiliation
[2] Novosibirsk State University, Novosibirsk, Russia
* Correspondence: kira.zadesenets@gmail.com

This PDF file includes:
Figures S1 to S9

A. RNA_8A

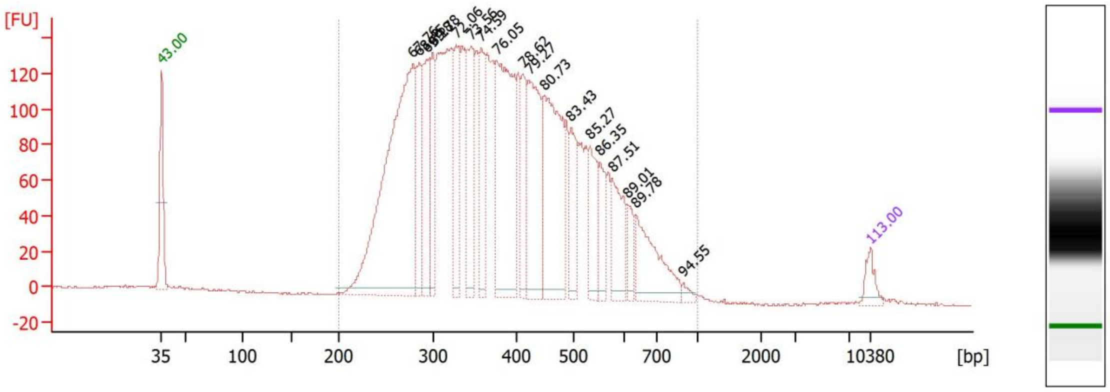

B. RNA_8C

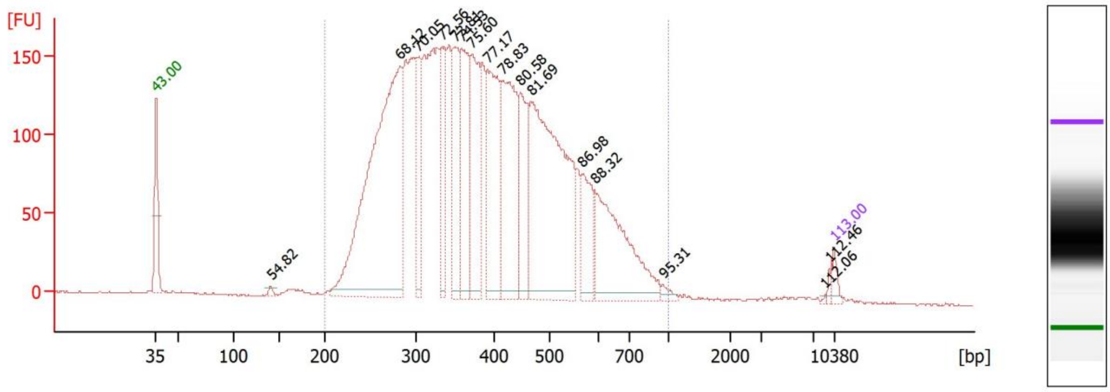

C. RNA_8E

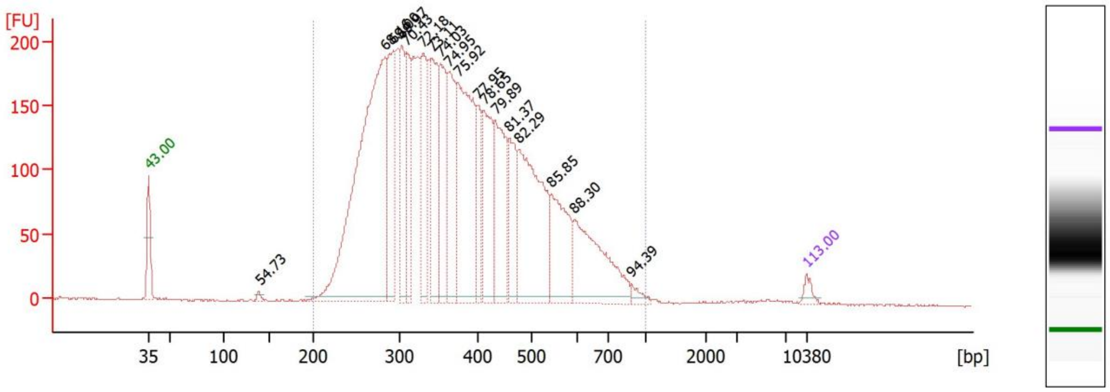

Figure S1. Electropherograms for the RNAseq libraries generated from the DV1_8 worm pools A, C, E.

A. RNA_10A

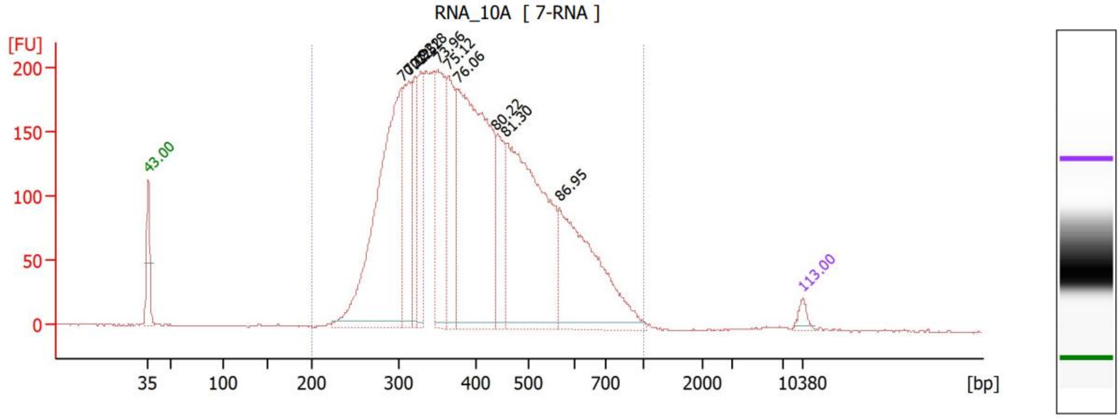

B. RNA_10C

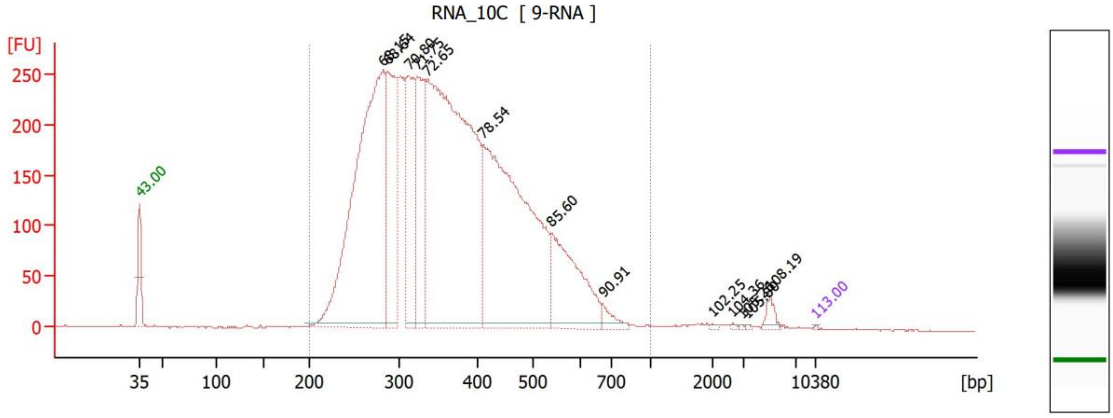

C. RNA_10E

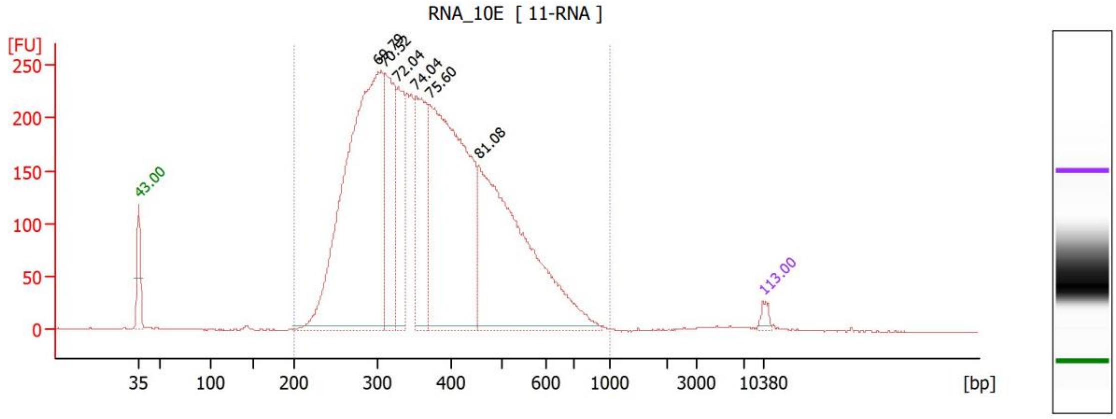

Figure S2. Electropherograms for the RNAseq libraries generated from the DV1_10 worm pools A, C, E.

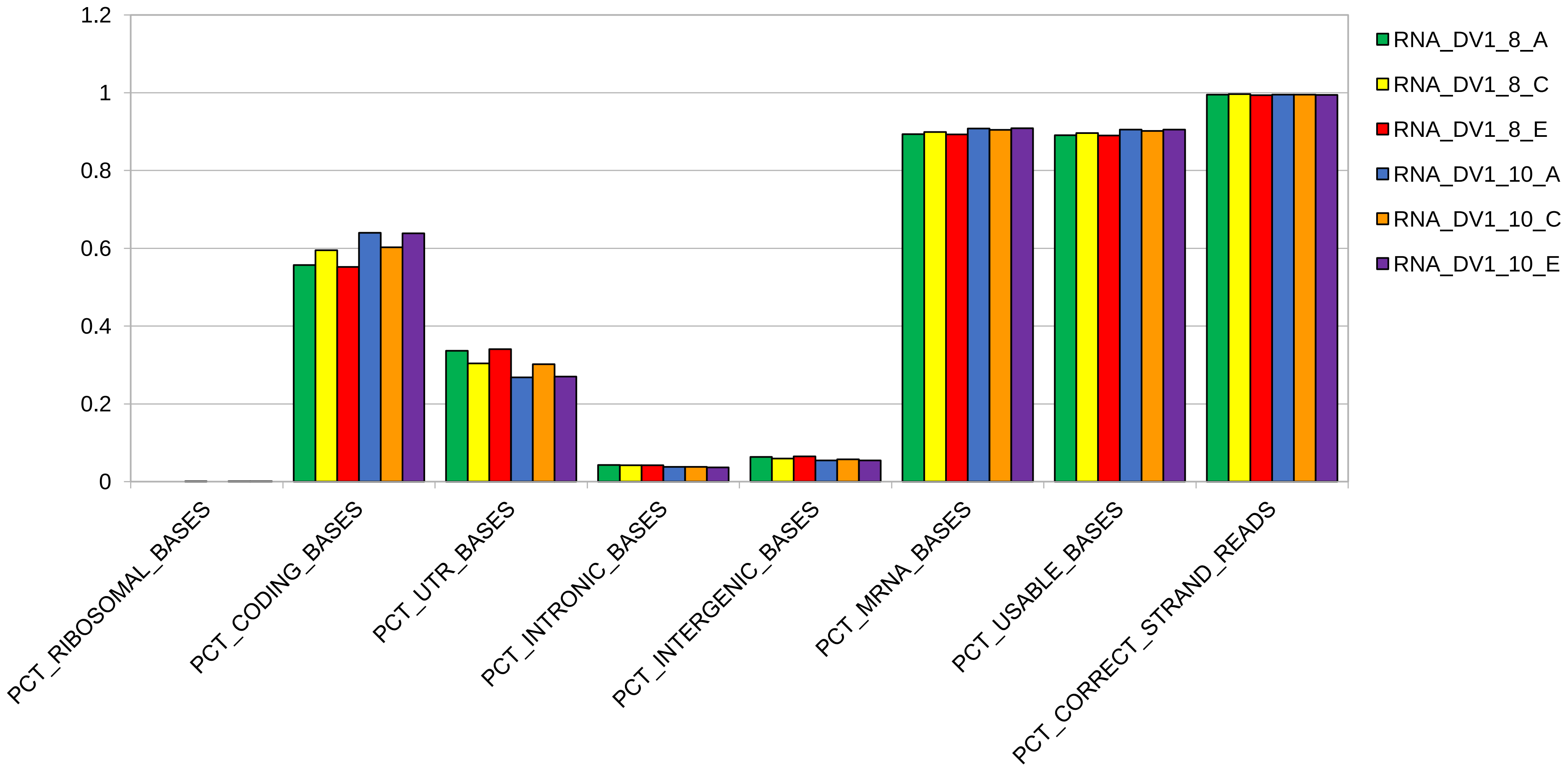

Figure S3. Metrics for quality assessment of the generated RNAseq libraries.

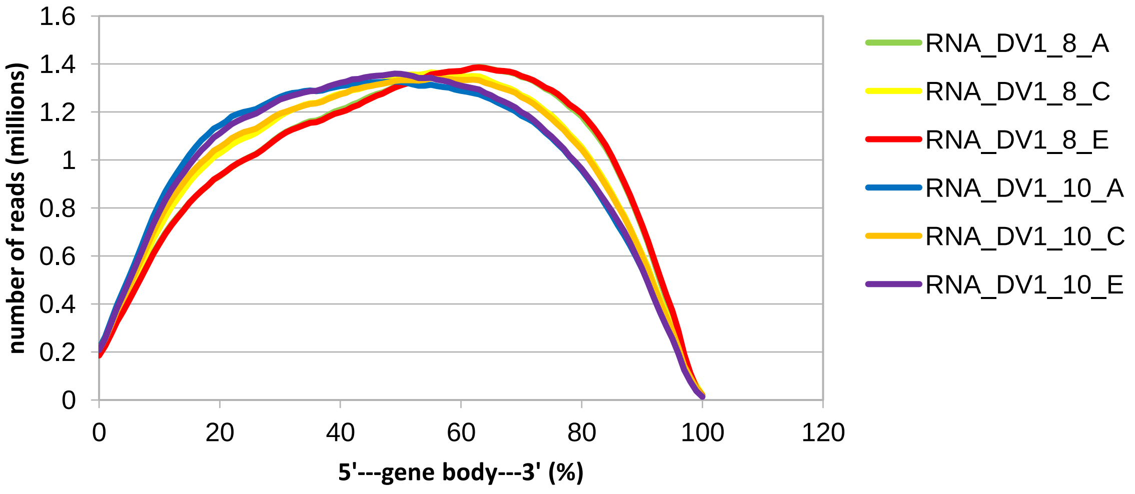

Figure S4. Gene body coverage on average for each RNAseq library. All libraries show even coverage. The percentages of the relative amount of reads that map closer to the 3' end than to the 5' end, i.e. the amount of reads that map to the right of the dashed vertical line. According to (Sigurgeirsson et al., 2014) quality assessment of the RNAseq libraries corresponds to RIN 8-10.

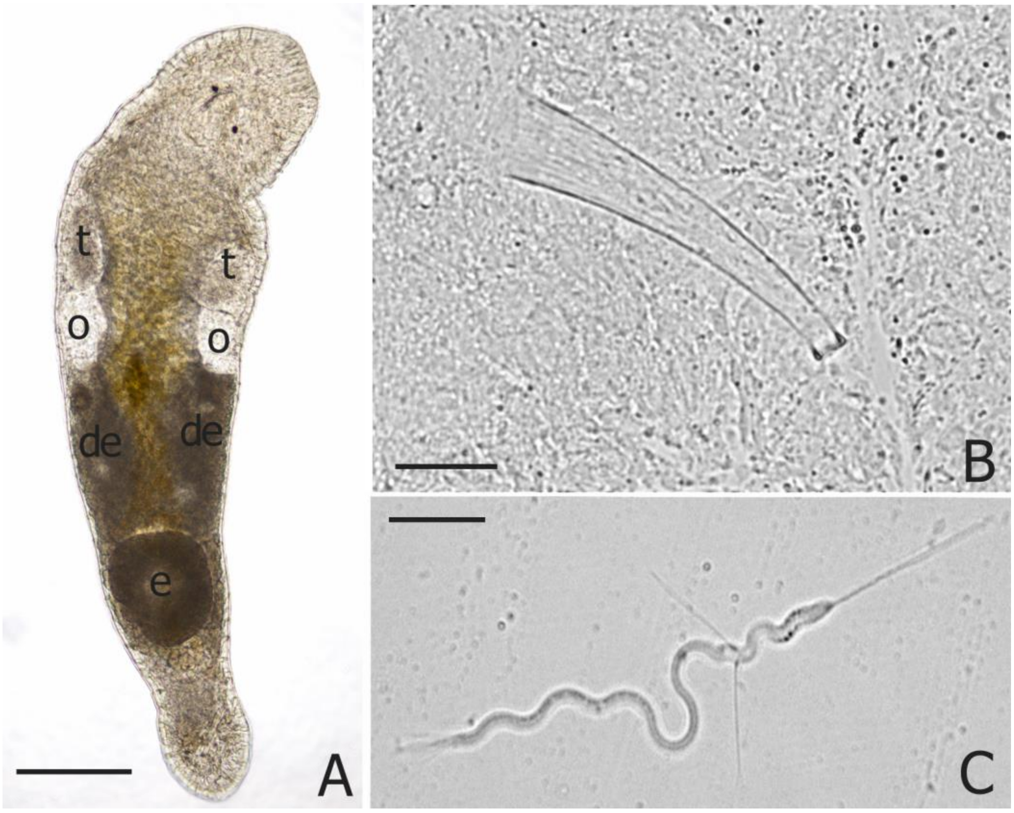

Figure S5. Transmission light microscopy of *M. lignano*. (A) the mature worm with visualized paired testes (t), ovaries (o), developing eggs (de) and egg (e), scale bar 100 mkm; (B) stylet, copulative male organ, scale bar 20 mkm; (C) sperm scale bar 10 mkm.

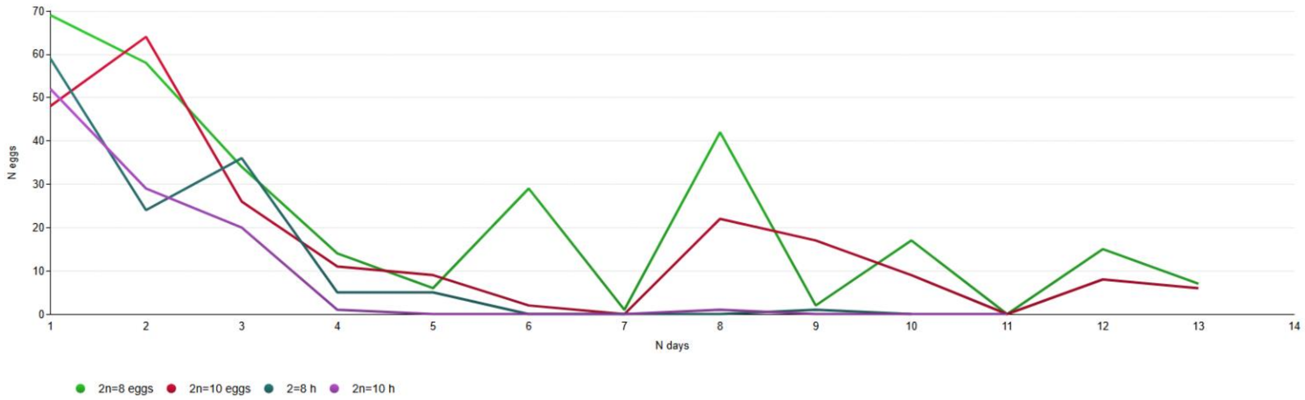

Figure S6. Dynamics of egg production and their hatching during experiment. Eggs laid by euploid (green color) and aneuploid (red color) worms, hatched eggs from euploids are marked dark blue color, from aneuploids in purple color.

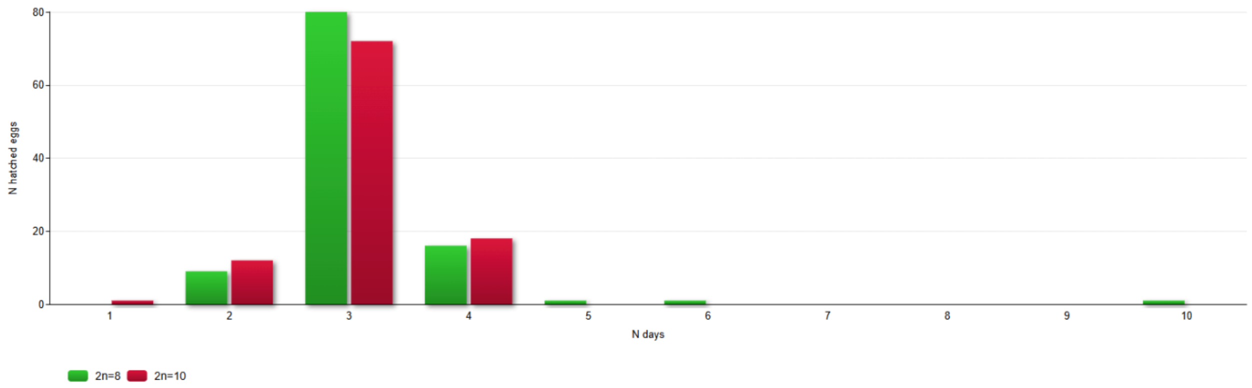

Figure S7. Development time from the egg to hatching for eu- and aneuploid worms.

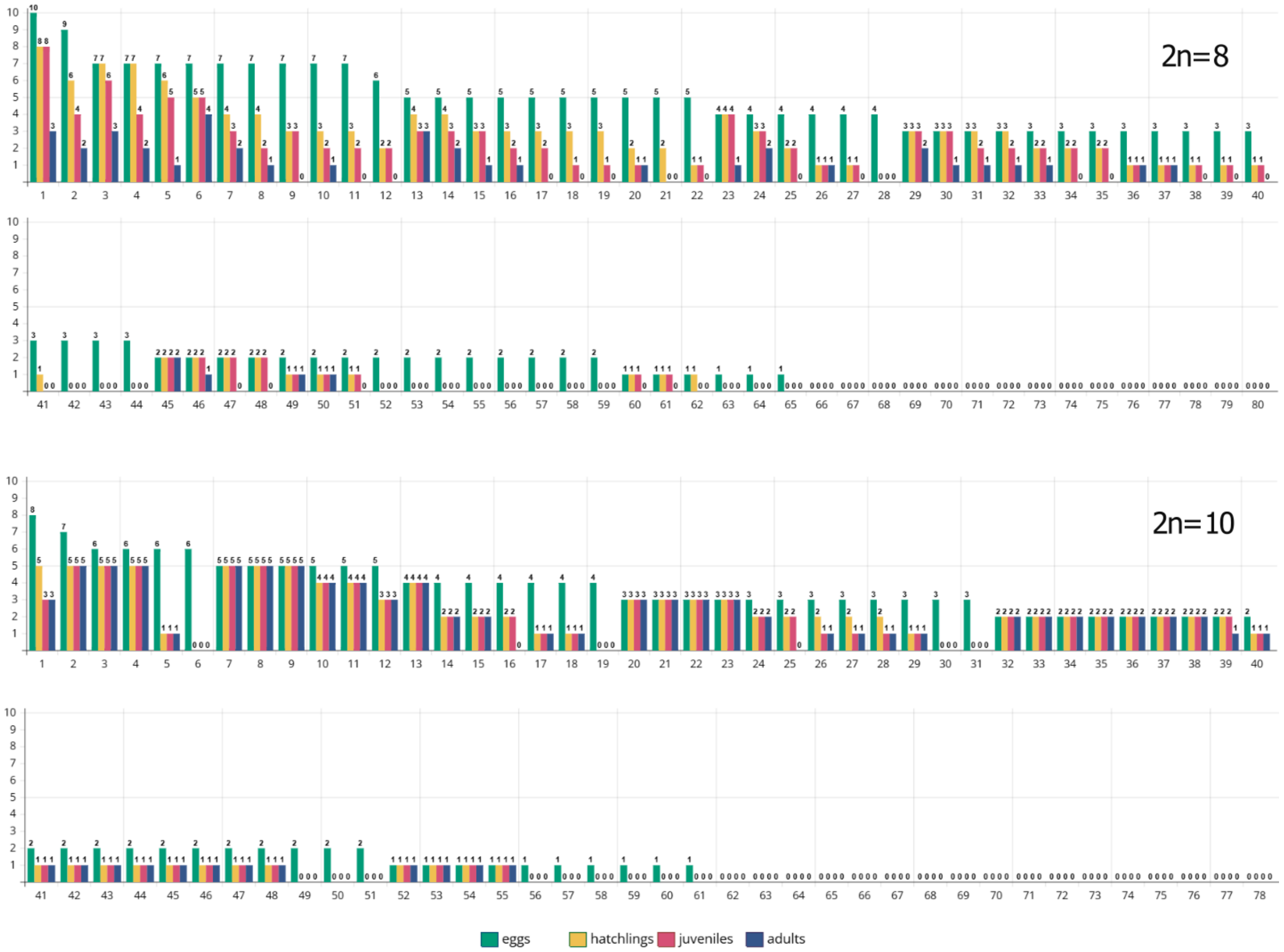

Figure S8. Fecundity of the the euploid (DV1_8) and aneuploid (DV1_10) *M. lignano* worms. The X-axis represents the ID of the worm. The number of laid eggs (green), hatchlings (yellow), juveniles (magenta), and matured worms (dark blue) from individual worm are marked on the top of each bar.

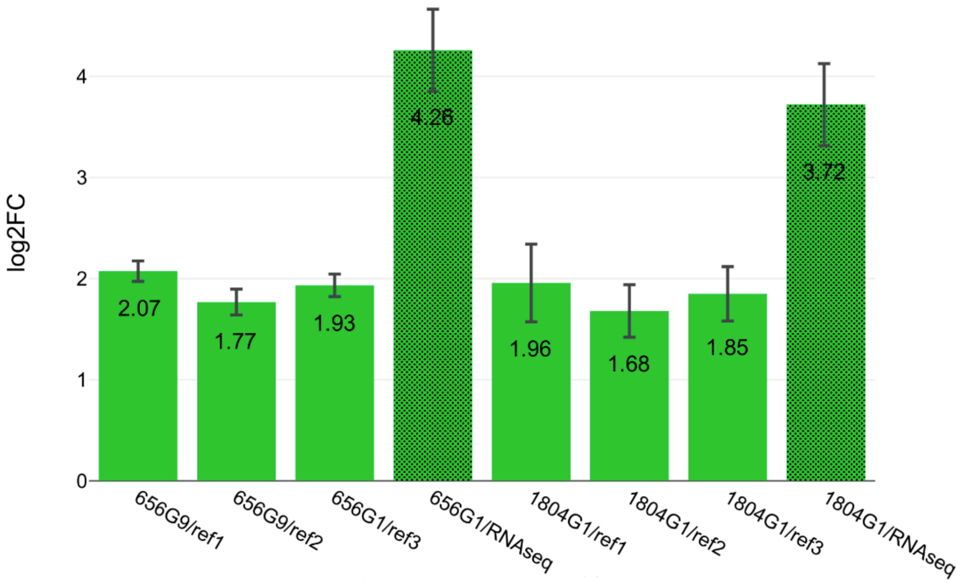

Figure S9. ddPCR validation for RNAseq data using DEGs of the DV1_10 subline. Each target transcript was normalized to the transcript copy number of the reference genes and calibrated to the worms of the DV1_8 subline. The data presented for the upregulated DEGs 656G9 and 1804g1. Ref1, 2, 3 correspondent to the reference genes C14804, C14077, and COX5B, correspondently.

References:

Sigurgeirsson, B.; Emanuelsson, O.; Lundeberg, J. Sequencing degraded RNA addressed by 3' tag counting. *PLoS One* **2014**, *9*, e91851. doi: 10.1371/journal.pone.0091851.
